# Bottleneck‐associated changes in the genomic landscape of genetic diversity in wild lynx populations

**DOI:** 10.1111/eva.13302

**Published:** 2021-10-08

**Authors:** Maria Lucena‐Perez, Daniel Kleinman‐Ruiz, Elena Marmesat, Alexander P. Saveljev, Krzysztof Schmidt, José A. Godoy

**Affiliations:** ^1^ Departamento de Ecología Integrativa Estación Biológica de Doñana (CSIC) Sevilla Spain; ^2^ Departamento de Genética Facultad de Biología Universidad Complutense Madrid Spain; ^3^ Department of Animal Ecology Russian Research Institute of Game Management and Fur Farming Kirov Russia; ^4^ Mammal Research Institute Polish Academy of Sciences Białowieża Poland

**Keywords:** bottleneck, endangered species, genetic diversity, genetic drift, genetic erosion, genetic load, genomic landscape, purifying selection

## Abstract

Demographic bottlenecks generally reduce genetic diversity through more intense genetic drift, but their net effect may vary along the genome due to the random nature of genetic drift and to local effects of recombination, mutation, and selection. Here, we analyzed the changes in genetic diversity following a bottleneck by comparing whole‐genome diversity patterns in populations with and without severe recent documented declines of Iberian (*Lynx pardinus*, *n* = 31) and Eurasian lynx (*Lynx lynx*, *n* = 29). As expected, overall genomic diversity correlated negatively with bottleneck intensity and/or duration. Correlations of genetic diversity with divergence, chromosome size, gene or functional site content, GC content, or recombination were observed in nonbottlenecked populations, but were weaker in bottlenecked populations. Also, functional features under intense purifying selection and the X chromosome showed an increase in the observed density of variants, even resulting in higher *θ*_W_ diversity than in nonbottlenecked populations. Increased diversity seems to be related to both a higher mutational input in those regions creating a large collection of low‐frequency variants, a few of which increase in frequency during the bottleneck to the point they become detectable with our limited sample, and the reduced efficacy of purifying selection, which affects not only protein structure and function but also the regulation of gene expression. The results of this study alert to the possible reduction of fitness and adaptive potential associated with the genomic erosion in regulatory elements. Further, the detection of a gain of diversity in ultra‐conserved elements can be used as a sensitive and easy‐to‐apply signature of genetic erosion in wild populations.

## 1 INTRODUCTION

Genetic diversity is one of the three main components of biodiversity, together with species and ecosystem diversity, which we need to preserve in the current biodiversity crisis. Genetic diversity is intimately related to adaptive potential and the loss of genetic diversity is generally associated with reduced fitness and increased extinction risks, so the importance of genetic variation transcends to species diversity and is also connected to ecosystem resilience (Allendorf et al., 2013). Despite this, genetic diversity is currently largely neglected in conservation legislation and practice and this has given rise to urgent calls to revert this situation and to incorporate the routine monitoring of genetic diversity, including wild species, in conservation programs (Des Roches et al., 2021; Hoban, Bruford, et al., 2021; Hoban, Campbell, et al., 2021; Laikre et al., 2020).

Genetic diversity in populations is the result of the interplay between mutation, genetic drift, recombination, selection, and gene flow (Ballenghien et al., 2017; Ellegren & Galtier, 2016). In demographically stable populations, the site frequency spectrum (SFS) and genetic diversity varies along the genome and across genomic features, following variations in the strength of recombination, selection and mutation, and the random action of genetic drift, creating a genomic landscape of genetic diversity. Changes in effective population size alter these equilibrium patterns of genetic diversity across the genome. In particular, a demographic bottleneck (i.e., a sudden reduction in *N*_e_) will increase the action of genetic drift and cause a general loss of genetic diversity, in a magnitude determined by its severity and duration (Garza & Williamson, 2001), and will distort the SFS through the preferential loss of low‐frequency alleles (Nei & Maruyama, 1975). However, we might expect that the effect of demographic bottlenecks on genetic diversity will locally depart from predictions based on equilibrium or simple drift models because evolutionary forces interact in complex ways between them and with genetic drift (Duret & Arndt, 2008; Duret & Galtier, 2009; Halldorsson et al., 2019; Pratto et al., 2014; Smith et al., 2018; Terekhanova et al., 2017; Williams et al., 2015).

Genetic diversity is ultimately created by mutation, and mutation rates, often estimated by divergence rates between species, vary extensively along and among chromosomes (Gonzalez‐Perez et al., 2019). For example, it is known that GC content is positively correlated with genetic diversity, partly due to the hyper‐mutability of CpG dinucleotides (Duret & Arndt, 2008; Duret & Galtier, 2009; Smith et al., 2018). Recombination is also positively correlated with genetic diversity due to its associated mutagenic effect (Duret & Arndt, 2008; Duret & Galtier, 2009; Halldorsson et al., 2019; Pratto et al., 2014; Smith et al., 2018; Terekhanova et al., 2017) and to its reduction of genetic hitchhiking and background selection, as described below (Stephan, 2010).

Natural selection impacts genetic diversity at selected sites by either reducing (purifying or negative selection, and positive selection) or maintaining it (balancing selection). The effects of selection on diversity may extend to neighboring linked neutral sites especially in regions of low recombination, a process often referred to as hitchhiking or linked selection. Selective sweeps and background selection are the consequence of linked positive and negative selection, respectively (Cutter & Payseur, 2013). Furthermore, the effectiveness of selection may be hampered if multiple linked loci experience selective pressures simultaneously via Hill‐Robertson interference (Felsenstein, 1974; Hill & Robertson, 1966).

A reduction in *N*_e_ would impact regions under selection leading to two predictions. Firstly, variants with selection coefficients lower than the inverse of *N*_e_ will behave as effectively neutral (Kimura, 1962), resulting in the accumulation of moderately deleterious variation in small populations due to the reduced efficacy of purifying selection (Charlesworth et al., 1993). Secondly, the higher inbreeding rates in small populations may expose in homozygosis the deleterious effects of (partially) recessive mutations, thus facilitating their elimination by selection (i.e., purging) (García‐Dorado, 2007; Garcia‐Dorado, 2012; Hedrick & Garcia‐Dorado, 2016).

Levels of genetic diversity vary extensively along and among chromosomes following variations in the relative intensity of these evolutionary processes; these correlations are often captured through proxies. For example, diversity is known to vary regionally with gene density, related to the action of natural selection (Martin et al., 2016; Payseur & Nachman, 2002), or with GC content, which correlates with mutation. Meanwhile, the low and high genetic diversity in centromeric and telomeric regions is largely attributed to low and high recombination rates, respectively. Sex chromosomes usually show highly contrasting patterns of diversity with respect to autosomes, as they are impacted quite differently by these processes. The X chromosome, for example, is expected to show ¾ of the diversity of the autosomes on the basis of its lower effective size due to male hemizygosity, but deviations from this ratio can arise due to several causes, including demographic changes (with bottlenecks reducing and population growth increasing the ratio; Pool & Nielsen, 2007), sex‐biased demography (e.g., reproductive success or migration rates), recombination only in females (excluding the pseudo‐autosomal region), sex‐biased mutation, and differences in selection regime due, among other things, to the exposure of variants in hemizygous males (reviewed in Schaffner, 2004; Wilson Sayres, 2018).

Genetic diversity has been traditionally assessed in wild species through the use of anonymous and purportedly neutral genetic markers, but genome‐wide assessments are becoming increasingly feasible and common (Hohenlohe et al., 2021). The transition from markers to genomes offers the opportunity to incorporate functional variation into the genetic assessment of endangered species, and this has prompted calls to abandon neutral variation in favor of functional variation and even to question the relevance of neutral diversity in conservation (Teixeira & Huber, 2021). Whereas there are many arguments and practical considerations for maintaining a focus on neutral variation in conservation genomic studies, it is clear that the field greatly needs a better understanding of the interaction of functional genetic diversity and demographic history (DeWoody et al., 2021; García‐Dorado & Caballero, 2021). Furthermore, the incorporation of functional variation might provide novel and much needed parameters to effectively detect populations where genetic diversity might be compromising fitness and population viability.

Despite the increasing interest and decreasing costs of genomic analyses in conservation, the lack of the required resources in most endangered species, such as a reference genome, and/or appropriate samples, has limited progress. We find, on one hand, few empirical studies assessing differences in diversity along the genome in stable populations of nonmodel organisms (e.g. Corcoran et al., 2017; Dutoit et al., 2017; Wang et al., 2016), and on the other, simulations exploring the dynamics of diversity in nonequilibrium scenarios, including demographic bottlenecks (Torres et al., 2020). To our knowledge, the few empirical studies analyzing patterns of diversity following a population bottleneck on wild conservation‐relevant populations mostly deal with genome‐wide averages, and at most contrast putatively neutral and selected coding sites (e.g., synonymous vs. nonsynonymous variation) (Grossen et al., 2020; Robinson et al., 2016, 2018; Saremi et al., 2019; Xue et al., 2015).

Here, we characterize the changes in the genomic landscape of diversity brought about by a demographic bottleneck in the two lynx species present in Eurasia, the highly endangered Iberian lynx (*Lynx pardinus*) and the broadly distributed Eurasian lynx (*Lynx lynx*). By taking advantage of the availability of a *de novo* assembled and annotated Iberian lynx reference draft genome (Abascal et al., 2016), we measure genetic diversity across chromosomes, chromosomal regions, and genomic features, in populations with contrasting recent demographic histories. To do so, we assess genomic diversity patterns in one population per species that best represent an ancestral unaffected (i.e., prebottleneck) scenario (from now on referred to as nonbottlenecked populations, NB), and we compare them with those of genetically eroded populations of the same lineage (bottlenecked populations, B), two populations of Eurasian lynx and one population of Iberian lynx. We also assess the role of recombination, mutation, and selection in shaping postbottleneck diversity patterns by comparing local values between regions or features with contrasting genetic diversity dynamics. By focusing on the variance of bottleneck effects across the genome, we aim to: (a) assess how the magnitude of loss of genetic diversity caused by a bottleneck varies among chromosomes, chromosomal regions, and genomic features, and (b) evaluate whether this magnitude is related to differences in local levels of selection, mutation, and recombination. By providing snapshots of the changes in genetic diversity in different functional categories, this study contributes to a better understanding of the fitness consequences of population bottlenecks and species declines. In particular, it alerts on the reduction of fitness and adaptive potential associated to the genomic erosion in regulatory elements, and proposes the gain of diversity in ultra‐conserved elements as a sensitive and easy‐to‐apply signature of genetic erosion.

## 2 MATERIALS AND METHODS

### 2.1 Study populations

The Iberian lynx was recognized as the most endangered felid in the world (Nowell & Jackson, 1996). In 2002, when the species was classified as critically endangered, only two populations persisted with <100 individuals in total, the only remnants of a likely panmictic population thousands of years ago, which became progressively contracted and fragmented in the last two or three centuries (Casas‐Marce et al., 2017; Ferreras et al., 2010; Palomares et al., 2011; Rodríguez & Delibes, 2003). The highly eroded and peripheral Doñana population remained isolated during two centuries at an estimated *N*_e_ of 20 individuals, reaching 10 in the last few decades (Casas‐Marce et al., 2013, 2017). In contrast, Andújar is the remnant of the Eastern Sierra Morena subpopulation, which remained large and genetically connected with other populations until ca. 1950, and has since decreased in size to a minimum of *N*_e_ ≈ 21 estimated for the year 2002 (Casas‐Marce et al., 2013, 2017) (see also Figure S4 in [35] for a demographic reconstructions of Iberian lynx populations from 1950 to 2015). This contrasting recent history is reflected in the overall genomic diversity in Doñana being about half of Andújar's, with the latter representing about 84% of that of the historical Iberian lynx metapopulation (Casas‐Marce et al., 2017).

Unlike the Iberian lynx, the Eurasian lynx is one of the most broadly distributed felids in the world (Breitenmoser et al., 2015). In Europe, there are large differences between populations in terms of neutral genetic differentiation and diversity (Lucena‐Perez et al., 2020). Particularly, NE‐Poland and the Scandinavian populations, represented here by Norway, went through pronounced declines and remained isolated from other lynx populations during the last century. Specifically, NE‐Poland and Norway went through sharp population declines 200–300 ya and maintained effective sizes below 100 for the last 100–150 years (Lucena‐Perez et al., 2020) (Bazzicalupo et al., 2021). On the contrary, eastern European populations remained well connected and relatively large, and Kirov represents the population of the European lineage with the highest genetic diversity (Lucena‐Perez et al., 2020; Ratkiewicz et al., 2014). This population has apparently maintained effective sizes well over 1,000 at least until 500 ya and over 500 until present. These contrasting histories are reflected in average genomic diversity, as reported previously, Poland and Norway showing around 80% and 74% of that found in Kirov, respectively (Lucena‐Perez et al., 2020).

For the sake of simplicity, and in light of their contrasting recent history, we refer to Doñana, Norway, and NE‐Poland as bottlenecked (B) populations, and Andújar and Kirov as nonbottlenecked (NB) populations, although the latter have also declined in size to a much lower extent.

### 2.2 Sampling, DNA extraction, library preparation, and sequencing

**FIGURE 1:**
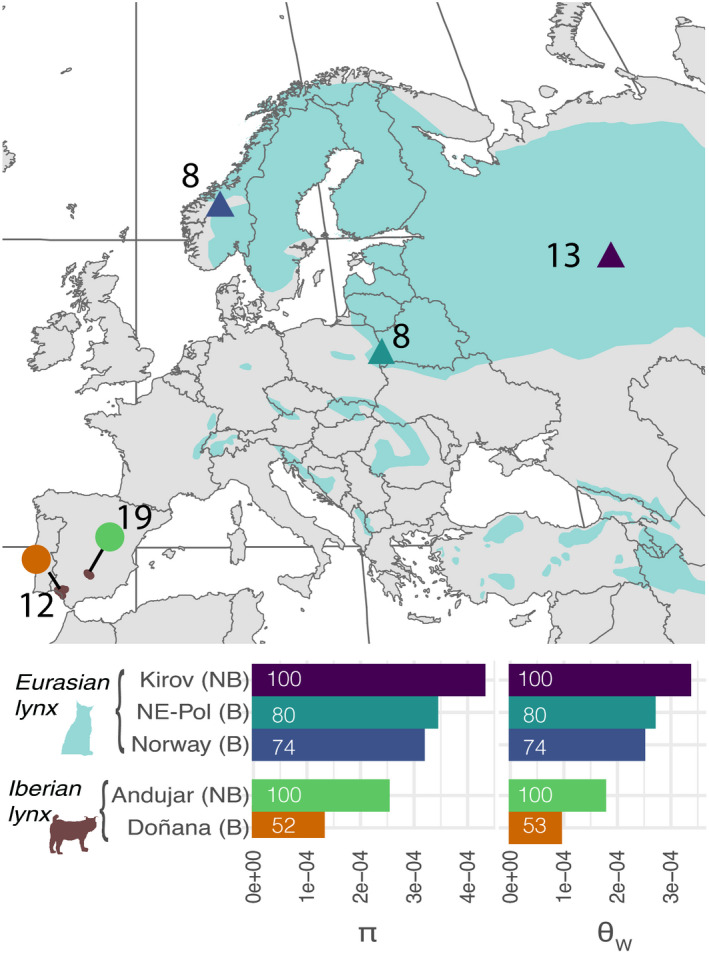
Sampling and global diversity (*π* and *θ*_W_) of the five studied populations. Sampled populations of the Eurasian lynx (triangle) and the Iberian lynx (circle) are marked within the distribution of the species (light blue for the European part of the Eurasian lynx distribution; dark red for the Iberian lynx). Diversity estimates for each population are represented below. For B populations, the diversity percentage relative to the reference NB population is indicated in the bar plot

Whole‐genome resequencing data were obtained from 60 Iberian and Eurasian lynx (Figure 1, Table S1). We sampled and processed 20 Iberian samples, which together with available sequence data from 11 additional individuals (Abascal et al., 2016), resulted in a total of 31 Iberian lynx from the two remnant populations: Andújar (*n* = 19) and Doñana (*n* = 12). We used 29 whole‐genome sequences from Eurasian lynx (Lucena‐Perez et al., 2020) from three different populations: Kirov region, Russia (*n* = 13), NE‐Poland (Białowieża and Knyszyn Primeval Forests) (*n* = 8), and Norway (*n* = 8). Samples were digested overnight using proteinase K and DNA was extracted using NucleoMag B‐beads (NucleoMag DNA from tissue kit) in LEM‐EBD facilities (Seville, Spain). gDNA samples were used for preparing Illumina sequencing compatible paired‐end libraries. The libraries were prepared, quantified, and sequenced in Illumina HiSeq2000 flowcell v3 (Illumina Inc.), in 2 × 101 bp paired‐end mode, following standard Illumina procedures at Centro Nacional de Análisis Genómico (CNAG‐CRG).

### 2.3 Quality control, trimming, and mapping

Iberian and Eurasian lynx data were quality‐controlled using FastQC (https://www.bioinformatics.babraham.ac.uk/projects/fastqc), and adaptors were removed when necessary using SeqPrep (https://github.com/jstjohn/SeqPrep). All short‐read sequence data were mapped to a 2.8 Gb Iberian lynx LYPA1.0 genome assembly (Abascal et al., 2016) using BWA‐MEM (Li, 2013) with default parameters. We added read groups to each sample using picard‐tools (https://broadinstitute.github.io/picard/) and merged the bam files from the same individual with SAMtools merge (Li et al., 2009). We marked PCR duplicates using picard‐tools (https://broadinstitute.github.io/picard/) and performed a local realignment and a base quality recalibration of the data using GATK 3.4 (McKenna et al., 2010). We calculated overall mapping stats using SAMtools flagstat (Li et al., 2009) and average depth using SAMtools depth (Li et al., 2009). We standardized sample depth to avoid any bias, so medium–high depth samples were subsampled to a depth within the range of the newly sequenced samples, using SAMtools view ‐s (Li et al., 2009). Average depth of coverage for Eurasian lynx samples was 5.9 x (range 5.0–7.6 x).

### 2.4 Genome annotation

The Iberian lynx reference genome has been annotated for several genomic features, namely, 3' UTR, CDS, introns, and 5' UTR (defined on the principal isoform of each gene), lncRNAs, and ncRNAs (Abascal et al., 2016). Here, we also included promoters of protein‐coding genes and lncRNA, defined as sequences 1000‐bp upstream of the gene or lncRNA, respectively, and Ultra Conserved Non‐Coding Elements (UCNEs). UCNEs (Dimitrieva & Bucher, 2013) were annotated by translating human coordinates (https://ccg.vital‐it.ch/UCNEbase/) into domestic cat Felis_catus_5.0 coordinates using LiftOver (http://rohsdb.cmb.usc.edu/GBshape/cgi‐bin/hgLiftOver), and these to lynx LYPA1.0 coordinates using lynx to cat synteny (Abascal et al., 2016). To define intergenic regions we added a security buffer of +/−1000 bp to any annotated region to avoid the influence of adjacent areas, using bedtools subtract and bedtools intersect (Quinlan & Hall, 2010). Each single 3' UTR, CDS, intron, 5' UTR, lncRNA, ncRNA, promoter (for genes and lncRNA), UCNE, and intergenic region—from now on, each single unit—was also assigned to chromosomes when possible using lynx to cat synteny, and excluding from the analysis units that overlapped more than one chromosome. We annotated as subtelomeric and pericentromeric those contigs containing more than 1000 bp syntenic to these regions in the domestic cat genome, that is, 2 Mb away from the telomeres and 10 Mb around the centromere, respectively (Abascal et al., 2016). Then, we calculated the percentage of sites in each unit overlapping subtelomeric, pericentromeric, or interstitial regions (defined as regions not pericentromeric or subtelomeric), and assigned units to regions where the overlap was ≥75%. The number of units, along with their average size, concatenated length, and number of total and informative sites, for each population, feature, and chromosome type is given in Table S12.

### 2.5 Genetic diversity per unit

For each population, genetic diversity per site was calculated using ANGSD (Korneliussen et al., 2013) with the following filters: −uniqueOnly 1 −remove_bads 1 −only_proper_pairs 1 −baq 1 −C 50 −minMapQ 30 −minQ 20 −doCounts 1 −minInd (number of individuals in the population/2) −setMaxDepth (average [AVR] depth for the population + [0.95*SD depth for the population]) −setMinDepth (AVR depth for the population − [0.95*SD depth for the population]) −skipTriallelic 1). First, we calculated the site allele frequency (SAF) and the site frequency spectrum (SFS) for each population using ANGSD and NGSTOOLS/realSFS (Korneliussen et al., 2013). This SFS plus its corresponding SAF were used to calculate nucleotide diversity (*π*), and Watterson theta estimator (*θ*_W_) per site (Korneliussen et al., 2013). It must be noted that ANGSD reports values of diversity per site whenever a site passes the filters, without distinguishing between variable and invariant sites, and that site diversity is not zero even for invariant sites because it considers the likelihood of all genotypes in the calculation. Then, we calculated diversity per unit by averaging diversity across sites, considering only informative sites (defined as all sites remaining after filtering). To avoid biases due to very few informative sites, we only considered units with more than 50 informative sites and with information for at least 20% of the positions. Results are presented for autosomes (A), unless otherwise noted. For the X chromosome, *θ*_W_ was corrected for the sample size of this chromosome based on the number of males and females sampled in each population (Lucena‐Perez et al., 2020). Additionally, we excluded the X chromosome pseudoautosomal region (PAR) delimited here as 10 Mb from the extreme of the chromosome, based on an estimate of 9 Mb for the domestic cat X chromosome PAR (Li et al., 2016).

We averaged *π*, and *θ*_W_ for the different populations, chromosomes, regions (subtelomeric regions vs. pericentromeric regions vs. interstitial), and features (Intergenic, Gen promoter, 5' UTR, CDS, Intron, 3' UTR, lncRNA promoter, lncRNA exons, lncRNA intron, ncRNA, UCNE), weighting by the number of informative sites. Then, we used *π*, and *θ*_W_ weighted means to calculate a measure of the skewness of the SFS toward rare variants (*S*) as 1−(*π*/*θ*_W_), where a positive *S* value indicates excess, and a negative value deficit, of rare variants over the mutation‐drift equilibrium for neutral alleles under the infinite sites model (Becher et al., 2020). Dispersion in diversity estimates (*SD*) was calculated by bootstrapping units using the R package boot (Canty & Ripley, 2017) with 100 iterations. We then computed ratios of diversity in B vs. NB populations in different chromosomes, regions, and features.

We also computed the ratios of *π*, and *θ*_W_ diversity between the X chromosome and the autosomes (X/A diversity ratios) for each population and feature. Dispersion in X/A ratios was calculated from dispersion in X and dispersion in A, using a propagation of the uncertainties formula, where *SD* refers to the standard deviation

$$\sqrt{\frac{A\;SD^{2}}{A^{2}}+\frac{X\;SD^{2}}{X^{2}}}*{\left(\frac{X}{A}\right)}^{2}$$

### 2.6 Relative diversity differences between B and NB populations across features

We quantified relative diversity differences between pairs of one B population and its corresponding NB population in different genomic features. By doing so, we take NB as the closest representation of the ancestral population and assume that observed differences in diversity are mostly due to population contraction in the B population. Comparisons included Norway vs. Kirov and NE‐Poland vs. Kirov for Eurasian lynx, and Doñana vs. Andújar for Iberian lynx. We used fix‐size adjacent windows on a concatenation of units of each feature to avoid distortions caused by differences in unit length across features. Additionally, we subsampled populations within species to the same number of individuals (i.e., eight for Eurasian, and 12 for Iberian lynx) to avoid biases due to differences in sample size.

We filtered concatenated sites for each population and feature within each species so that it contained the same positions in the two populations being compared, and computed diversity for each population and feature in nonoverlapping 1 kb windows. We calculated differences in diversity over these nonoverlapping 1 kb windows to be able to compute statistical tests without the bias introduced by different features having different average length. Relative differences in diversity (*δ*) were computed as:

$$\frac{\text{B diversity}‐\text{NB diversity}}{\text{B diversity}+\text{NB diversity}}.$$

In comparison with a direct diversity ratio (B diversity/NB diversity), this expression reduces the occurrence of the denominator being zero, shows a reduced variance, is bound to values between −1 and 1, and shows an approximately symmetrical distribution around the mean within the bounds. A positive *δ* means that the B population shows more diversity than the NB population and vice versa. Window diversity, computed as the arithmetic mean of site diversities estimated by ANGSD taking into account genotype uncertainty, is never zero. As a consequence, the distribution of the genetic diversity in windows shows a clearly bimodal distribution, with the lowest mode (usually 10^−5^ to 10^−7^) corresponding to windows with probably no diversity (Figure S1). As the diversity estimated for these windows with no diversity might still differ in the two populations, rendering irrelevant and probably biased *δ* values, we transformed *δ*_θW_ and *δ*π to zero for windows where the diversity in both populations was below the empirically determined diversity threshold separating the two modes (Figure S1). We plotted *δ*, and its dispersion calculated by bootstrapping over windows as implemented in Hmisc package in R (Harrell Jr, 2019) with 1,000 iterations. Also, in order to directly compare the magnitude of the changes in the density of variable sites with changes in their allele frequencies, we plotted *δ*_θW_ against *δ*_π_. Due to non‐normality of the *δ* statistic revealed by Shapiro–Wilk normality test, we compared the *δ* distribution among different features using a Wilcoxon signed‐rank test for paired samples and applied a strict Bonferroni correction for multiple tests to assess significance. All tests were run in R as implemented in stats package.

### 2.7 Genomic variables

We first annotated units for the following genomic variables: average recombination rate, divergence, as a proxy of mutation rate, GC content, and two different scores related to selection strength ( Residual Variation Intolerance Score (RVIS), and functional site percentage). Secondly, nonoverlapping 1 kb windows used for calculating diversity differences were also annotated for these genomic variables. We did this by averaging the values annotated for the units included in each 1 kb window weighting by the number of overlapping sites.

To calculate recombination rate, we used the latest domestic cat linkage map (Li et al., 2016). Original coordinates, which are referred to Felis_catus_8.0 assembly, were first translated to Felis_catus_5.0 and then to LYPA1.0 coordinates using lynx to cat synteny (Abascal et al., 2016). We assigned to each unit the average recombination rate of the 2 MB window where it was included. Divergence was computed as the number of observed substitutions between the Iberian or the Eurasian lynx and the bobcat (*L*. *rufus*), divided by the number of informative sites in each unit, as reported by Lucena‐Perez et al. (2020). To estimate GC content per unit, we used the Iberian lynx LYPA1.0 genome assembly, or a consensus Eurasian lynx genome (Abascal et al., 2016). Using BEDTools nuc (Quinlan & Hall, 2010), we parsed a bed file with units coordinates and estimated the GC percentage of each unit. The level of tolerance to variation of each gene was estimated by RVIS, a gene‐based score computed for human sequence data that reaches more negative values for intolerant genes (Petrovski et al., 2013). Lynx genes were annotated using a lynx‐to‐human orthologs database (Abascal et al., 2016). For functional sites percentage, we annotated the genome based on 2 Mb windows. For each 2 Mb window, we summed up the number of sites belonging to 3' UTR, CDS, 5' UTR, lncRNAs, ncRNAs, promoters, or UCNEs, divided it by 2 Mb and multiplied it by 100. Windows were then intersected with units using bedtools intersect (Quinlan & Hall, 2010).

### 2.8 Testing relationships between genetic diversity and genomic variables and differences in genomic variables between windows with contrasting behavior

To test how the relationships between different variables—namely chromosome size, gene content, recombination, divergence, GC content, RVIS, or functional sites percentage—and genomic diversity variables in the populations considered, we did linear regressions between those variables and genetic diversity at the chromosome level for each population. Chromosome length, gene content, and recombination rates were obtained from domestic cat reference genome, downloaded from https://www.ncbi.nlm.nih.gov/genome/?term=txid9685[orgn].

Then, for each of the three pairwise NB‐B comparisons we analyzed the linear regression of genetic diversity in the NB population with genetic diversity in the corresponding B population, separately for centromeric, interstitial, and telomeric regions. We report both the predictor (i.e., the slope) and the proportion of variance explained (*R* ^2^) as heat maps.

We then focused on genic—CDS and introns—and intergenic regions to assess whether there are significant differences between windows that showed higher diversity in B than NB population (*δ*_θW_ > 0.1) and the rest of the windows in the average values of the following genomic variables: NB diversity (*π*, *θ*_W_, and *S*) as a proxy of the ancestral diversity, recombination rate, RVIS and functional sites percentage as a proxy of selection, divergence as proxy of mutation rate, and GC content. After rejecting normality of these variables of interest, we used a Wilcoxon signed‐rank test for unpaired samples. The significance threshold was adjusted by applying a strict Bonferroni correction. To get an estimation of the effect of these variables, we calculated effect size (*r*), as implemented in (Mangiafico, 2020). Next, for each NB‐B pairwise comparison we focused on the subset of windows with no *θ*_W_ diversity in the NB population, and compared windows with no *θ*_W_ diversity in the B population either, that is, no diversity in NB and no diversity in B windows (ND_NB_‐ND_B_ windows), against those with some *θ*_W_ diversity in the B population (ND_NB_‐D_B_ windows) (Figure S1). Again, we used a Wilcoxon signed‐rank test for unpaired samples and Bonferroni correction to assess significant differences. All statistics were calculated using R (R Core Team, 2019), and results were graphed using ggplot2 R package (Wickham, 2009).

## 3 RESULTS

### 3.1 Bottlenecks reduce average genomic diversity

As a first approach to genomic diversity patterns, we estimated genome‐wide averages across populations and species. Their comparison between NB and B populations provides an overall indication of the relative intensity and duration of bottlenecks in the different populations. At the species level, the Eurasian lynx shows about twice the genome‐wide diversity of the Iberian lynx (Eurasian lynx: *π* weighted mean (wm) = 3.3*10^−4^; *θ*_Wwm_ = 2.8*10^−4^); Iberian lynx *π*_wm_ = 1.9*10^−4^; *θ*_Wwm_ = 1.4*10^−4^) (Figure 1). Both species are among the mammals with the lowest overall genomic diversity reported so far (Abascal et al., 2016; Lucena‐Perez et al., 2020). Within Eurasian lynx, whole‐genome diversity statistics are *π*_wm_ = 3.4*10^−4^ and *θ*_Wwm_ = 3.3*10^−4^ in the NB Kirov population, and these are reduced to 80% and 74% in the B NE‐Poland and Norway populations, respectively, for both *π* and *θ*_W_ (Figure 1, Table S2). In Iberian lynx, both *π* and *θ*_W_ diversity in the Doñana B population are ~52% of that found in the NB Andújar population (*π*_wm_ = 2.5*10^−4^; *θ*_Wwm_ = 1.8*10^−4^) (Figure 1, Table S2). Based on the relative diversity of each B population with respect to their NB population counterpart, bottlenecks rank from less to more extreme (longer duration and/or smaller *N*_e_) in the order: NE‐Poland, Norway, and Doñana. All populations show a negative *S* (an indication of a generalized scarcity of rare alleles) (Becher et al., 2020), particularly the Iberian lynx ones (average across populations of *S* = −0.41 vs. *S* = −0.28 in Eurasian lynx) (Table S2).

### 3.2 Bottlenecks lead to a larger accumulation of low‐frequency variants in the X chromosome than in the autosomes

**FIGURE 2:**
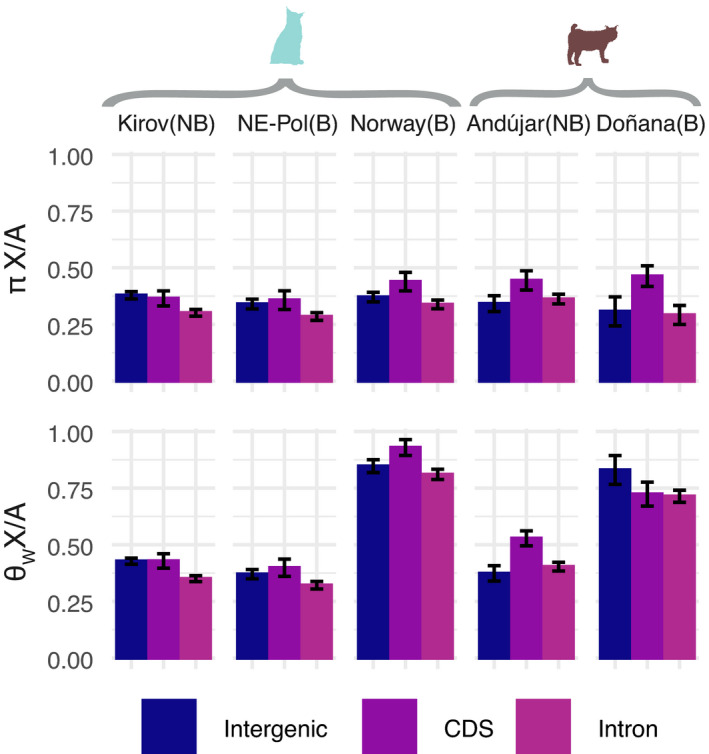
X/A ratio for *π* and *θ*_W_ diversity for the five populations analyzed, and three different genomic features (CDS, introns, and intergenic)

We then compared genetic diversity across chromosomes in NB and B populations, with a particular focus in the comparison of autosomes to the X chromosome. Chromosomes differ widely in diversity within populations (Figure S2; Table S4). Largest diversity differences occur between any of the autosomes (A) and the X chromosome, with the latter showing lower diversity than A consistently across populations (Figure S2; Tables S3 and S4). Global X/A ratios range across populations from 0.31 to 0.37 for *π*_X_/*π*_A_, and from 0.36 to 0.84 for *θ*_WX_/*θ*_WA_ (Table S3). While *π*_X_/*π*_A_ is similar to *θ*_WX_/*θ*_WA_ in NB populations and NE‐Poland, *θ*_WX_/*θ*_WA_ is notably larger than *π*_X_/*π*_A_ in the B Doñana and Norway populations (Figure 2). This pattern is consistent across different genomic features, namely, CDS, intergenic, and introns (Figure 2). Also, the diversity reduction in B relative to NB populations is globally similar for A and X (*π*_B_X_/*π*_NB_X_ is similar to *π*_B_A_/*π*_NB_A_); however, the larger (above 1) *θ*_WB_X_/*θ*_WNB_X_ than *θ*_WB_A_/*θ*_WNB_A_ for Norway and Doñana, when compared to Kirov and Andújar, respectively, indicates a larger density of variants in the X chromosome in B relative to NB populations. Accordingly, *S* values are negative in the NB Andújar and Kirov, and also in the B NE‐Poland populations, but positive in the B Doñana and Norway populations (Table S2).

### 3.3 Bottlenecks blur expected differences in diversity among and along autosomes

In order to better understand the consequences of bottlenecks in the patterns of genetic variation, we estimated the level of correlation of the genetic diversity in different autosomes to several known genomic correlates, and compared them in NB and B populations. Among the autosomes, average chromosomal genetic diversity is positively related to divergence, GC content, gene content, functional sites percentage, RVIS (a gene‐based measure of the deficit of standing functional variation, used here as a proxy for the intensity of ongoing purifying selection), and recombination, and negatively related to chromosome size in all populations of both species (Figure S3, Table S4). However, these correlations show lower predictors and smaller fractions of explained variance (*R* ^2^) in the B populations, especially so for *θ*_W_ (Figure S3).

To assess the contribution of recombination to changes in genetic diversity following a bottleneck, we compared genetic diversity in NB and B populations in different chromosomal regions that differ widely in recombination rates. Along autosomes, subtelomeric regions show the highest diversity, followed by the interstitial regions, and lastly the pericentromeric regions (Figure S4) in all populations. Subtelomeric regions show 1.52–1.66, and 1.29–1.62 times the interstitial *π* and *θ*_W_ diversity, respectively, with lower ratios of *θ*_W_ occurring in the B Norway and Doñana populations, indicating that *θ*_W_ diversity in B pops is still higher in subtelomeric regions than in other chromosomal regions, but not as higher as in NB populations (Table S5). Pericentromeric regions show 0.83–0.94 and 0.87–0.94 of interstitial *π* and *θ*_W_ diversity, respectively, both ratios tending to be higher in B populations than in their NB counterparts. Skewness of SFS is moderate and similar across chromosomal regions in NB populations, but the more extremely bottlenecked populations show more negative *S* values in subtelomeric regions, indicating a relatively larger overall scarcity of low‐frequency alleles.

Diversity across chromosomal regions ranks according to recombination rates (subtelomeric = 3.7 cM/Mb, interstitial = 3.5 M/Mb, and pericentromeric = 2.3 cM/Mb), and divergence (subtelomeric = 7.9e‐03, interstitial = 5.5e‐03, and pericentromeric = 5.2e‐03), and inversely to functional sites percentage (subtelomeric = 3.3, interstitial = 6.0, and pericentromeric = 6.3). Also, subtelomeric regions show higher GC content (subtelomeric = 0.58, interstitial = 0.47, and pericentromeric = 0.48), and higher RVIS (subtelomeric = 0.2, interstitial = −0.31, and pericentromeric = −0.28) (Table S5).

Finally, we assessed the level of correlation between autosomal genetic diversity in NB with that of their respective B populations. Autosomal diversities in NB and B populations were generally correlated, but the more intense the bottleneck, the weaker these correlations became both in terms of predictor and *R* ^2^ (Figure S5), reflecting the random effects of genetic drift. In addition, these correlations were weaker in subtelomeric regions compared to pericentromeric or interstitial regions (Figure S5).

### 3.4 Smaller relative diversity reductions in B populations in selectively constrained features

**FIGURE 3:**
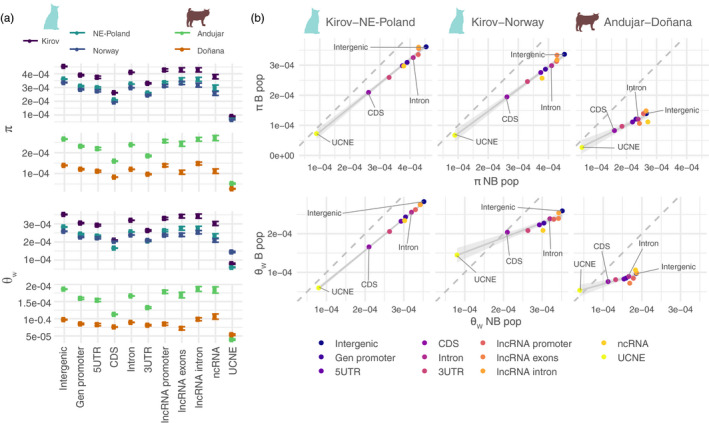
(a) Average *θ*_W_ and *π* diversity for different features in nonbottlenecked (NB: Kirov and Andújar) and bottlenecked (B: Norway, NE‐Poland, Doñana) populations of Eurasian lynx (upper subpanel) and Iberian lynx (lower subpanel). Error bars represent the standard deviation (*SD*) obtained from bootstrapping. (b) Relationship between diversity (*θ*_W_ or *π*) of the NB (*X* axis) vs. B populations (*Y* axis) for different features. The dashed line represents the diagonal, where the diversity of NB would be equal to B. Diversity is generally lower in B populations than NB populations across features, but the difference is smaller for selected features (e. g. CDS) and is even reverted in UCNE in two out of the three comparisons

To explore the influence of natural selection on the changes in genetic diversity occurring in B populations, we compared genetic diversity in NB and B populations across genomic features that differ in biological function and their expected level of purifying selection. For all the features considered, namely, intergenic regions, coding gene promoters, 5' UTR, CDS, introns, 3' UTR, long‐non‐coding RNA promoters, long‐non‐coding RNA exons, long‐non‐coding RNA introns, non‐coding RNA (mostly miRNAs, snRNAs, and snoRNAs), and ultra‐conserved‐non‐coding‐elements (UCNE), patterns of genetic diversity are negatively correlated with their anticipated selective pressure (Figure 3, Table S6). Taking intergenic as a reference, the most selectively constrained features present the lowest diversity, in particular UCNE (0.19–0.20 and 0.21–0.56 of intergenic *π* and *θ*_W_, respectively), followed by CDS (*π* = 0.58–0.6; *θ*_W_ = 0.59–0.79; both relative to intergenic). With regard to the NB‐B comparisons, regulatory regions such as gene promoters, noncoding RNAs, but also 5' UTR and 3' UTR, and even introns, show smaller reductions of diversity in B populations with respect to NB than intergenic regions. *S* is negative for all populations and features, except for UCNE in Norway and Doñana, which shows a high positive value (*S* = 0.53 and *S* = 0.50, respectively), and for CDS in Norway (*S* = 0.04), suggesting an accumulation of low‐frequency variants in these constrained features in the most bottlenecked populations (Table S6). Notably, despite the lower diversity in B than in NB populations in most features, diversity in UCNE in the B Doñana and Norway populations is actually higher than in their reference NB population (Andújar and Kirov, respectively) when measured as Watterson's theta (*θ*_W_), but not as nucleotide diversity (*π*) (Figure 3).

**FIGURE 4:**
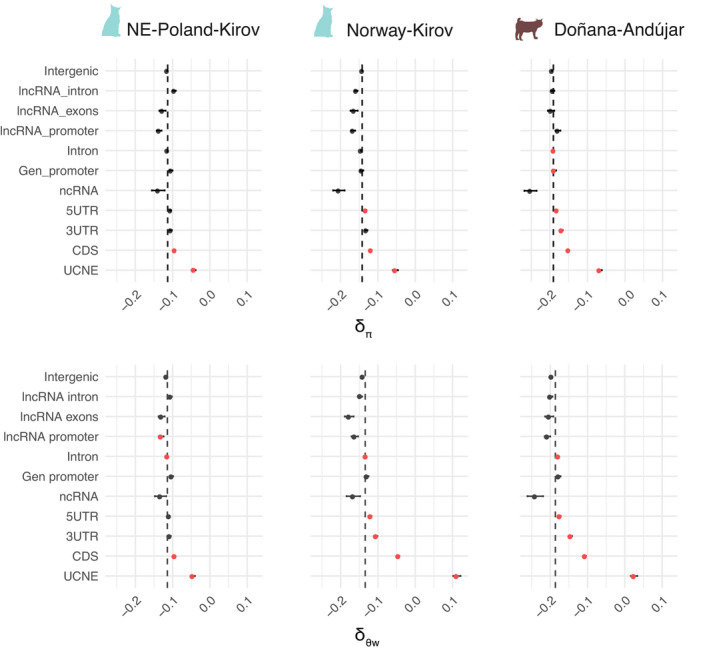
*δ*_θW_ and *δ*_π_ across features for the three comparisons of B vs. NB populations. Positive values indicate higher diversity in the B population. The dashed lines represent the average *δ* value considering all features. Errors bars are the *SD* obtained from bootstrapping the data. The colored circle, triangles, and squares represent significant *p*‐values when compared to intergenic after Bonferroni correction (Table S7)

Sliding window analysis of relative diversity differences between NB and B populations (*δ*) shows the lower overall diversity in the B population as a negative global mean of *δ* for most features, both for *π* (*δ*_π_) and *θ*_W_ (*δ*_θW_) (Figure 4). For intergenic regions, Doñana‐Andújar shows the more extreme diversity difference, followed by Norway‐Kirov, and finally NE‐Poland‐Kirov. Regarding other features, and consistently among comparisons, *δ*_θW_ and *δ*_π_ for UCNE are less negative, and *δ*_θW_ becomes even positive for the Doñana‐Andújar and Norway‐Kirov comparisons (Figure 4). A similar trend of less negative *δ* values is also noticeable for CDS, and more subtly for 3' UTR, and 5' UTR, indicating again a smaller diversity loss for selectively constrained genomic features (relative to putatively neutral ones) in bottlenecked populations (Figure 4).

*δ*_θW_ and *δ*_π_ are generally correlated, but *δ*_θW_ tends to be less negative than *δ*_π_ for most features and in the most bottlenecked populations (Doñana and Norway). This pattern is particularly notable for UCNE followed by CDS, 3' UTR and 5' UTR. Although with lower *δ* than intergenic, ncRNA *δ*_θW_ tends to be larger than *δ*_π_ in the Norway‐Kirov comparison, and to a lesser extent in the Doñana‐Andújar comparison (Figure S6).

### 3.5 Gains in diversity in B populations occur in regions of higher mutational input and in genes more tolerant to variants

**FIGURE 5:**
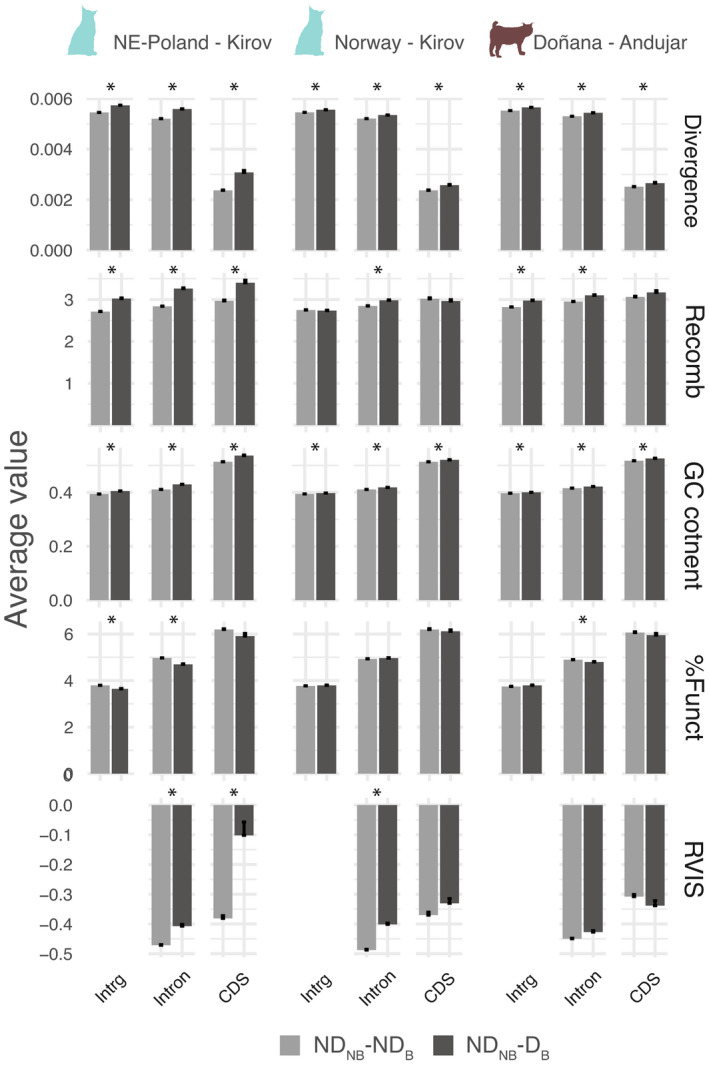
Comparison of average values of different genomic variables in windows with no diversity in both NB and B populations (ND_NB_‐ND_B_ windows) vs. windows with no diversity in NB but some diversity in B populations (ND_NB_‐D_B_ windows). The asterisk represents significant differences between both sets of windows. ND_NB_‐D_B_ windows tend to be in regions with higher recombination rate, divergence, and content. They also tend to be associated with genes more tolerant to changes in the moderate, but not in the most extreme bottlenecks

In order to gain further insights into the processes driving diversity gains in B populations, we compared average values of different genomic variables between windows of the same feature that show net *θ*_W_ diversity gains with windows that do not. Windows with *δ*_θW_ > 0.1, that is, with larger *θ*_W_ in B than in NB populations, show significantly lower diversity in NB populations (*π*, *θ*_W_) and larger *S*, that is, SFS more skewed toward rare alleles, than the rest of windows (*p*‐value < 3e‐04; strict Bonferroni correction; Figure S7, Table S8), the effect sizes of these variables measured as a correlation coefficient (*r*) being larger for Norway‐Kirov (range: |0.15|–|0.22|), followed by Doñana‐Andújar (range: |0.09|–|0.14|), and finally NE‐Poland‐Kirov comparison (|0.01|–|0.04|) (Table S9). They also show significant differences in other genomic variables like divergence, recombination, or selective constraints, which often are opposite to that expected from their effect in diversity (Figure S7, Table S8). For instance, windows with *δ*_θW_ > 0.1 (i. e. which gain diversity after the bottleneck) tend to show lower diversity in the corresponding NB population, but are in regions with higher divergence, GC content, and recombination, which are generally associated to high diversity. Consequently, the magnitude and significance of these contrasts become larger when we limit the set of windows being considered to those showing no diversity in the NB population, that is, when we compare windows that lack *θ*_W_ diversity in both NB and B populations (ND_NB_‐ND_B_ windows) to those with no diversity in NB but with some diversity in the B population (ND_NB_‐D_B_ windows) (Figure 5, Tables S10 and S11). Therefore, ND_NB_‐D_B_ windows tend to be in regions with higher divergence and GC content than ND_NB_‐ND_B_ windows. Also, recombination rate is significantly higher in ND_NB_‐D_B_ windows, for nonselectively constrained features in the Norway‐Kirov (introns), and Doñana‐Andújar comparisons (intergenic and introns). Regarding selection, coding, but also intronic, ND_NB_‐D_B_ windows tend to be in genes with higher RVIS, that is, more tolerant to change, in moderate NB‐B pairwise comparisons, but not so in the most extreme Doñana‐Andújar comparison. More generally, differences in genomic variables between ND_NB_‐ND_B_ and ND_NB_‐D_B_ windows are smaller overall when the bottleneck is more intense (Norway and Doñana) and the features are selectively constrained (CDS).

## 4 DISCUSSION

Here, we present one of the most comprehensive empirical studies of the genomic consequences of bottlenecks in natural populations. By directly comparing genomic diversity in Eurasian and Iberian lynx populations with contrasting recent demography, we assessed how the impact of recent bottlenecks on population genetic diversity varies across chromosomes, chromosomal regions, and genomic features. Also, we explored how different genomic variables, such as mutation and selection, as well as the initial SFS, might be affecting the different outcomes of genetic drift and, in consequence, the changes in diversity following a demographic decline. Our study provides empirical evidences of the conspicuous action of drift across the genome in replicated natural bottleneck scenarios. Comparing three bottlenecks of different intensity allowed us to assess the consistency of the results across replicates, and to account for differences regarding drift intensity.

Recent bottlenecks are expected to globally reduce the genomic diversity in a magnitude determined by the intensity and duration of the bottleneck, and our estimates grossly rank according to records and demographic reconstructions from genetic and genomic data (Abascal et al., 2016; Casas‐Marce et al., 2017; Lucena‐Perez et al., 2020). Skewness of the SFS is negative in all populations, indicating a general scarcity of low‐frequency variants, which is however more pronounced in the Iberian lynx populations, consistent with the expectation for its smaller effective size at mutation‐drift equilibrium.

One major finding of our study is that the magnitude of diversity reductions occurring in bottlenecked populations varies extensively across chromosomes, chromosomal regions, and genomic features. One first consequence of this is that correlations between the genetic diversities in NB and B populations across chromosomes and chromosomal regions weaken as bottleneck intensity increases, especially in subtelomeric regions. Also, the correlations of genetic diversity with genomic variables like recombination, divergence, or gene content, which are expected at migration‐mutation‐selection‐drift equilibrium and still observed in the NB populations, also weaken or disappear in the B populations. Both observations indicate that, in B populations, the reduction in mean diversity is accompanied by an increased variance across chromosomes and chromosomal regions, making genetic diversity patterns to progressively depart from those expected at equilibrium. Whereas the stochastic nature of genetic drift is a major contributor to the increased variance in genetic diversity in bottlenecked populations, our results also suggest an important contribution of its interaction with mutation and selection, as discussed below.

Regarding the X chromosome and autosomes comparison, results suggest a larger accumulation of low‐frequency alleles in X relative to A (X/A ratios) in B populations. The X chromosome differs from the A in their response to a bottleneck because: (a) its effective size is ¾ of A, which predicts a ratio of diversity X/A of 0.75 at mutation‐drift equilibrium; (b) it has a lower recombination rate; (c) it has a lower mutation rate; and (d) recessive deleterious variation is continuously exposed to selection in hemizygous individuals (i.e., males in mammals) (Arbiza et al., 2014). Besides, under population size change scenarios, the dynamics of the two types of chromosomes might differ, with X diversity undergoing faster changes than autosomal diversity (Pool & Nielsen, 2007). It is unclear which of these distinctive characteristics is responsible for the observed marked accumulation of low‐frequency variants in X in B populations. Although its lower effective size will translate to a larger number of selected sites behaving as neutral, the fact that the pattern is observed also at neutral regions suggests processes other than relaxed purifying selection are involved.

An accumulation of low‐frequency alleles is also observed in some selectively constrained features, such as CDS and UCNE in the autosomes. Features that are subject to unequal selective pressures behave very differently in populations that underwent different degrees of population size decline. Whereas some features, like intergenic regions, ncRNA and promoters show a similar relative reduction of *θ*_W_ and *π* diversity in B populations, others such as UCNE and CDS and, to a lesser extent, 5' and 3' UTR, show a relatively smaller reduction in *θ*_W_ than in *π*, which in the extreme case of UCNE results in higher *θ*_W_ (suggestive of an excess of low‐frequency variants) in the B than in the corresponding NB populations. Such increase in *θ*_W_ in selectively constrained features is, in principle, unexpected from purely random processes, since random genetic drift in bottlenecked populations would result in the preferential loss of alleles in the low‐frequency side of the spectrum, where most selectively constrained alleles will be (Nei, 1975). The pattern observed for the X chromosome and some selectively constrained features could thus be the consequence of (a) methodological limitations related to limited sampling, (b) various evolutionary processes other than genetic drift, like mutation and selection, acting by themselves or through their interaction with drift.

Regarding methodological limitations, it must be noted that the estimates of genetic diversity, and especially *θ*_W_ diversity, are conditioned by both the sample sizes and the SFS. Given our sample sizes of 16 and 24 genomes, alleles segregating at frequencies below the detection threshold will be underrepresented in our samples. One consequence is that their likely loss in bottlenecked populations will remain largely unnoticed, whereas a few haplotypes increasing in frequency by drift will result in an increase in the observed *θ*_W_ diversity. This is especially true for those regions and features with the highest skew in SFS, that is, the most selectively constrained features like UCNE and CDS. On the other hand, the possibility of observing an increase in *θ*_W_ diversity following a bottleneck will also depend on the SFS and thus on starting *π* diversity. Indeed, we found that windows with higher diversity tend to lose diversity whereas those with low diversity are more likely to gain diversity after the bottleneck. Interestingly, windows that gain diversity also tend to show higher divergence and GC content, which could be directly related to a higher mutational input generating a larger number of alleles segregating at low frequencies in the prebottleneck population and favoring the accumulation of de novo mutations during the bottleneck. Given the relatively short separation of the populations (i.e., likely less than 60 generations in the case of the Eurasian lynx (Lucena‐Perez et al., 2020), and around 40 generations in Iberian lynx (Casas‐Marce et al., 2017), assuming a generation time of 5 years (Lucena‐Perez et al., 2018)), and their small effective size (in the range of tens), it is unlikely that a large fraction of the observed accumulation of variants at low frequencies in the B populations is due to *de novo* mutations since the start of the bottleneck. Thus, most of these alleles increasing in frequency during and after the bottleneck would be pre‐existing variants that remain undetected before the bottleneck and which increase in frequency and are thus recorded and result in higher observed *θ*_W_ after the bottleneck. Recombination could also contribute to this pattern through its direct mutagenic effect (Duret & Arndt, 2008; Duret & Galtier, 2009; Halldorsson et al., 2019; Pratto et al., 2014; Smith et al., 2018; Terekhanova et al., 2017; Williams et al., 2015).

The accumulation of additive deleterious variants due to the relaxation of purifying selection could also be contributing to the observed increase in *θ*_W_ in selectively constrained regions (Balick et al., 2015; Kirkpatrick & Jarne, 2000). Empirical evidence for this has been found in domesticated species (Makino et al., 2018; Marsden et al., 2016), while evidence in humans remains controversial (Do et al., 2015; Henn et al., 2016; Lohmueller, 2014; Simons & Sella, 2016; Simons et al., 2014). The reduced efficacy of purifying selection in bottlenecked populations would affect those variants with 1/2*N*_e_NB_ < *s* < 1/2*N*_e_B_, that is, alleles with *s* between these values would be efficiently selected in the NB but will drift in frequency as neutral variants in the B population; for example, in the case of Andújar (*N*_e_ = 20) and Doñana (*N*_e_ = 10) the corresponding range would be 0.025 < *s* < 0.05, which are rather high values. Moreover, it must be noted that while Doñana has remained at such low size for many decades, Andújar was much larger a few generations ago, so the range of mutations that behave as neutral in Doñana and not in Andújar may be substantially wider (with a lower inferior *s* limit), so that the actual number of variants involved may be relatively large (Casas‐Marce et al., 2017). The contribution of the reduced efficacy of purifying selection is further supported by the observation that in shallower demographic declines the smaller relative loss of diversity occurs mainly in genes with higher RVIS (i.e., more tolerant to changes). However, under more extreme bottlenecks the relationship with RVIS weakens and becomes nonsignificant, suggesting that the relaxation of purifying selection results in the accumulation of potentially deleterious variants even in highly intolerant genes, and alerting of possible fitness reductions. Finally, empirical and theoretical studies show that selectively constrained diversity approaches a new equilibrium faster than neutral diversity (Brandvain & Wright, 2016; Gordo & Dionisio, 2005; Pennings et al., 2014; Song & Steinrücken, 2012). We can thus expect that the diversity in CDS and UCNE that is due to additive mutations reaches its new increased equilibrium level faster than intergenic or intronic diversity reaches their new reduced equilibrium level.

### 4.1 Possible fitness consequences for the B populations

Our results suggest that overall diversity reductions in bottlenecked populations affect regulatory elements, such as promoters, or ncRNA, in a similar fashion as intergenic regions. This loss of diversity is likely removing pre‐existing variation in the regulation of gene expression. The loss of variation in regulatory elements of transcription likely compromises these populations’ potential for acclimation to the new environments through phenotypic plasticity, reducing their viability under environmental fluctuations. Furthermore, there is increasing evidence that regulatory variation can be an important source of adaptations, possibly a more important source than changes in protein sequence for rapid evolution, so its loss can have a major impact on adaptive potential (Harrisson et al., 2014). These observations call for greater attention to regulatory variation and patterns of gene expression in conservation genomics.

For other selectively constrained features, our results suggest that overall genetic diversity reductions in bottlenecked populations are accompanied by the accumulation of possible deleterious alleles in functional regions such as CDS, including genes highly intolerant to changes (in the most extreme bottlenecks), and extending beyond coding regions to include UCNE, and to a lesser extent 5' UTR and 3' UTR. The latter two features are essential for efficient transcription and for the post‐transcriptional regulation of gene expression, and variants in both regions have been linked to several diseases (Hindorff et al., 2009). Regarding UCNE, an accumulation of deleterious variation in these regions is likely to affect their prominent function as regulatory *cis* elements, especially during development (Marcovitz et al., 2016; Polychronopoulos et al., 2017), leading to a reduction in fitness.

Our results call for extending the analyses of genetic variation in endangered species to include functional variation beyond coding sequences. Including regulatory elements will broaden our understanding of the effects of drift on the genome and allow for a more comprehensive assessment of the possible short‐term fitness reductions and the long‐term loss of adaptive potential in endangered species. As the genome‐wide assessment of genetic variation in nonmodel species is becoming increasingly feasible, and more readily facilitated by the increasing availability of reference genomes, the widespread implementation of genetic monitoring in wild species demands simple and cost‐effective methods, and informative and easy to implement indicator variables. In this regard, the accumulation of variants in highly constrained UCNEs may provide an easily detectable and reliable signal of recent demographic declines.

## Supporting information

Fig S1‐S7

Table S1‐S12

## Data Availability

The data that support the findings of this study are openly available in European Nucleotide Archive (ENA) at https://www.ebi.ac.uk/ena, accession PRJEB44874.
