# Supplementary material for: Bottleneck‐associated changes in the genomic landscape of genetic diversity in wild lynx populations
Source: Evol Appl. 2021 Oct 8;14(11):2664–79. doi: 10.1111/eva.13302 (PMC8591332; doi:10.1111/eva.13302)
Supplement: Supplementary file 1 — Fig S1‐S7 [file EVA-14-2664-s001.pdf]

Bottlenecked-associated changes in the genomic landscape of genetic diversity in wild lynx populations

Supplementary Figures

Fig. S1. B vs NB diversity plot and quadrant definition.

Fig. S2. Average θ_W and π diversity for different chromosomes.

Fig. S3. Heatmap representing correlations between different genomic variables and θ_W and π diversity for the five lynx populations studied.

Fig. S4. Average θ_W and π diversity for different chromosomal regions.

Fig. S5. Heatmap representing correlations between the diversity in B and NB populations in different genomic regions.

Fig. S6. Relationship between $\delta_{\theta W}$ *vs.* δ_{π} for each feature for different B-NB comparisons.

Fig. S7. Average values for different genomic variables for windows with $\delta_{\theta W} > 0.1$ *vs.* windows with $\delta_{\theta W} < 0.1$.

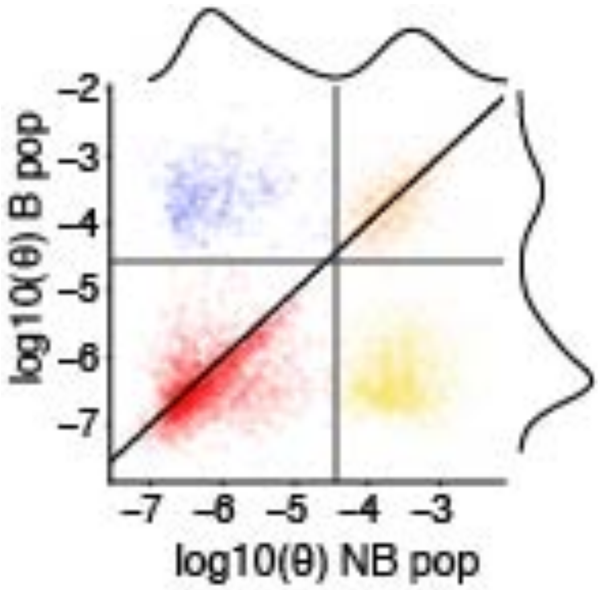

Fig. S1. B vs NB diversity plot and quadrant definition. The distribution of window diversity within populations, as estimated by ANGSD, is markedly bimodal with one mode corresponding to units that show almost no diversity (i.e. ~10^{-6}), and a second mode, with units that do hold some diversity (i.e. ~10^{-4}). Therefore, in a graph plotting diversity of B *vs.* NB we can define four quadrants: units with no diversity in both populations (ND-ND) *vs.* units with no diversity in the NB population but showing diversity in the B population (ND-D), units that show diversity in both populations (D-D), and units that show diversity in the NB population but no diversity in the B one (D-ND). For some of our analyses we compare the value of several genomic variables between windows in ND-ND quadrants versus windows in the ND-D quadrants. In order to avoid biases introduced by differences across datasets in the empirical diversities estimated for otherwise invariable sites, we manually assign them a diversity of zero when calculating delta statistics.

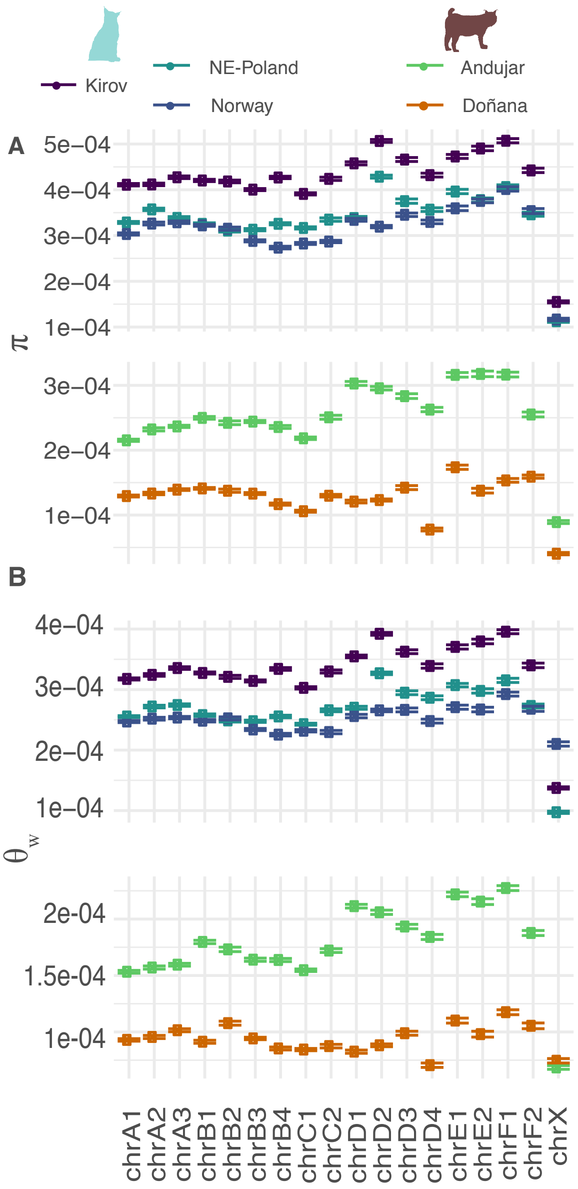

Fig. S2. Average θW and π diversity for different chromosomes. Error bars are standard deviation (stdev) obtained from bootstrapping.

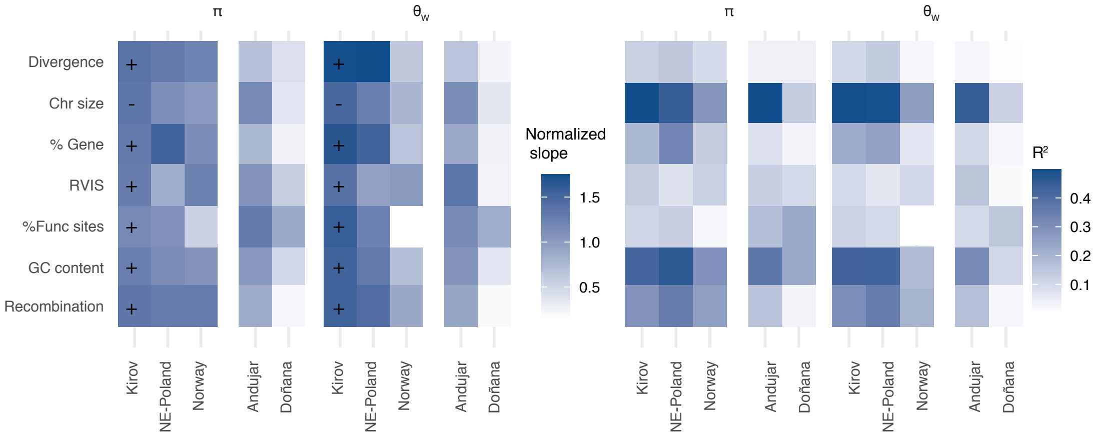

Fig. S3. Heatmap representing correlations between different genomic variables and θ_W and π diversity for the five lynx populations studied. On the left the slope of the linear regression normalized across variables for comparison purposes across populations (normalized slope=slope value / mean slope value across populations). On the right R^2 values.

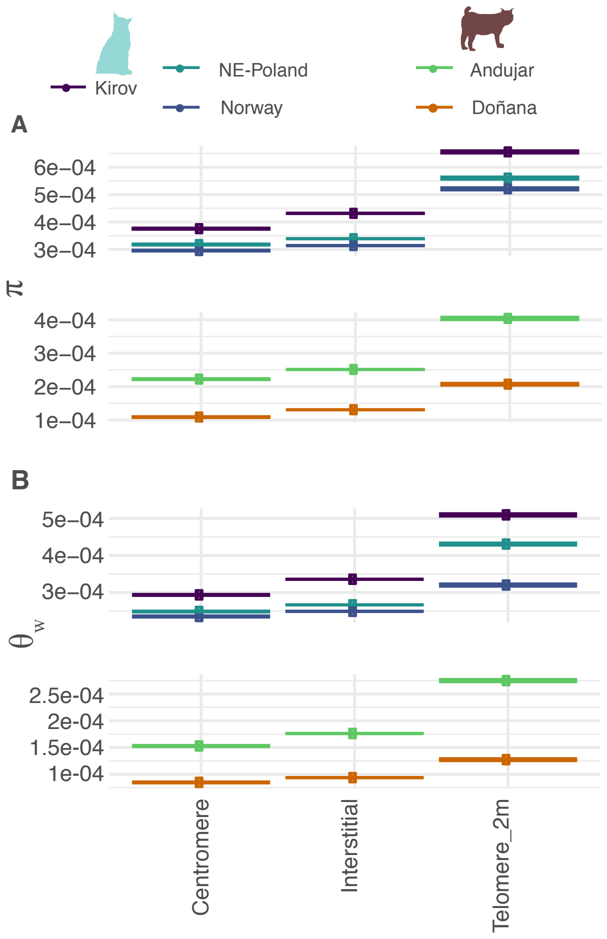

Fig. S4. Average θ_W and π diversity for different chromosomal regions. Error bars are standard deviation (stdev) obtained from bootstrapping.

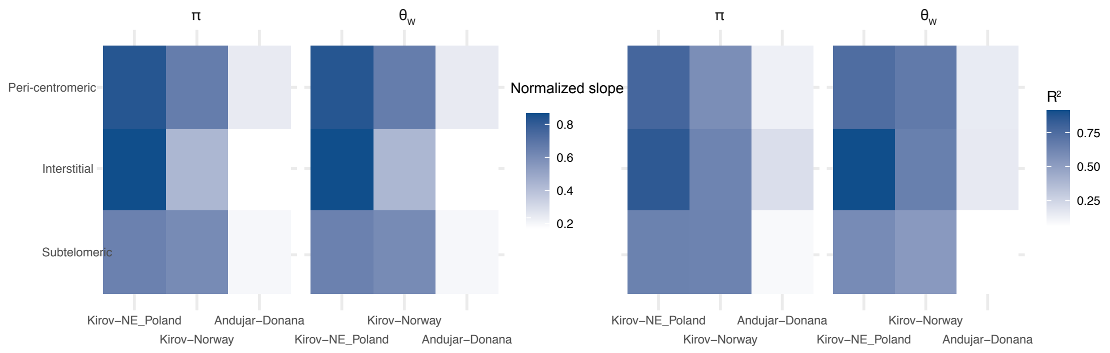

Fig. S5. Heatmap representing correlations between the diversity in B and NB populations in different genomic regions. On the left the slope of the linear regression normalized across variables for comparison purposes across populations (normalized slope=slope value / mean slope value across populations). On the right, the corresponding R^2 values.

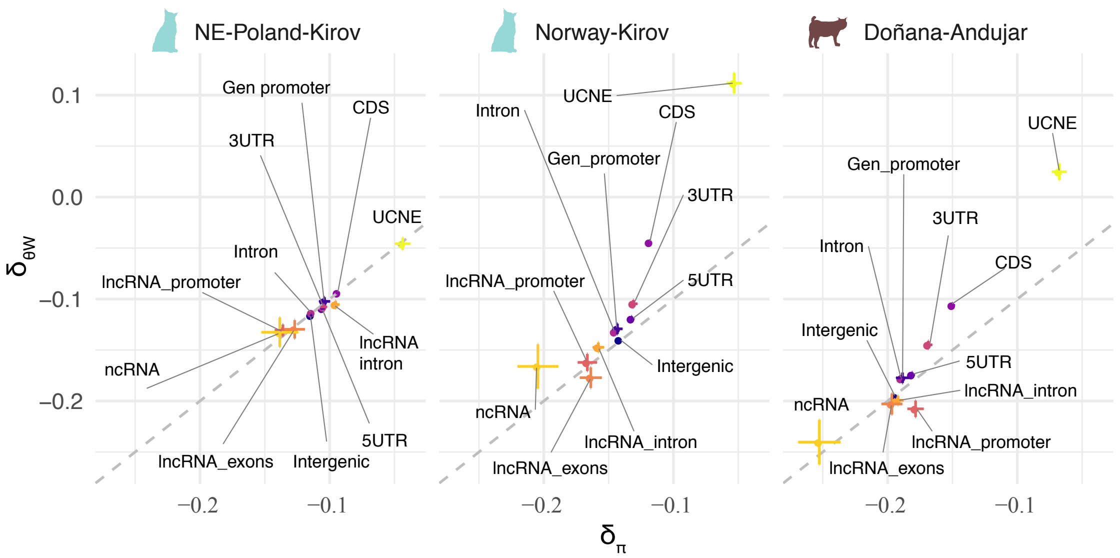

Fig. S6. Relationship between $\delta_{\theta W}$ *vs.* δ_{π} for each feature for different B-NB comparisons.

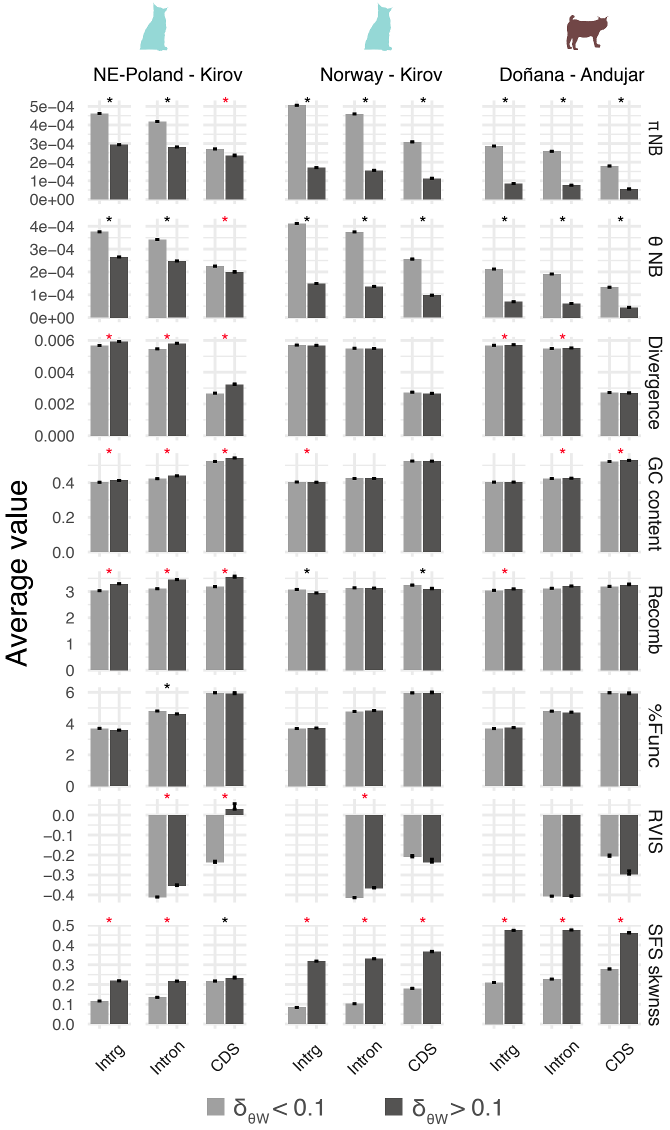

Fig. S7. Average values for different genomic variables for windows with $\delta_{\theta W} > 0.1$ *vs.* windows with $\delta_{\theta W} < 0.1$. Black asterisk represents significant differences between both categories where $\delta_{\theta W} < 0.1$ is significantly higher, while red asterisk represents the opposite. Note that even though the mean value could be higher, sometimes the test is significant for the opposite direction.
